# Supplementation with Fermented Rice Bran Attenuates Muscle Atrophy in a Diabetic Rat Model

**DOI:** 10.3390/nu12082409

**Published:** 2020-08-12

**Authors:** Tubagus Bahtiar Rusbana, Afifah Zahra Agista, Wahyu Dwi Saputra, Yusuke Ohsaki, Kouichi Watanabe, Ardy Ardiansyah, Slamet Budijanto, Takuya Koseki, Hisashi Aso, Michio Komai, Hitoshi Shirakawa

**Affiliations:** 1 Laboratory of Nutrition, Graduate School of Agricultural Science, Tohoku University, 468-1 Aramaki Aza Aoba, Aoba-ku, Sendai 980-8572, Japan; tubagus.bahtiar.rusbana.t8@dc.tohoku.ac.jp (T.B.R.); agista@g-mail.tohoku-university.jp (A.Z.A.); wahyu@g-mail.tohoku-university.jp (W.D.S.); yusuke.ohsaki.a4@tohoku.ac.jp (Y.O.); mkomai@m.tohoku.ac.jp (M.K.); 2 Department of Food Technology, Faculty of Agriculture, University of Sultan Ageng Tirtayasa, Serang 42110, Indonesia; 3 Cellular Biology Laboratory, Graduate School of Agricultural Science, Tohoku University, 468-1 Aramaki Aza Aoba, Aoba-ku, Sendai 980-8572, Japan; watakoh@tohoku.ac.jp (K.W.); asosan@tohoku.ac.jp (H.A.); 4 International Education and Research Center for Food Agricultural Immunology, Graduate School of Agricultural Science, Tohoku University, 468-1 Aramaki Aza Aoba, Aoba-ku, Sendai 980-8572, Japan; 5 Department of Food Technology, Universitas Bakrie, Jakarta 12920, Indonesia; ardiansyah@bakrie.ac.id; 6 Faculty of Agricultural Engineering and Technology, IPB University, Bogor 16680, Indonesia; slamet.budijanto@gmail.com; 7 Faculty of Agriculture, Yamagata University, Tsuruoka 997-8555, Japan; tkoseki@tds1.tr.yamagata-u.ac.jp

**Keywords:** fermented rice bran, muscle atrophy, diabetes mellitus, streptozotocin, FBXO32/Atrogin-1, TRIM63/MuRF1, NF-κB, anti-inflammation

## Abstract

Fermented rice bran (FRB), a prospective supplement, has been proven to ameliorate certain medical conditions. However, its nutraceutical effect on muscle atrophy has never been investigated. The present study aimed to evaluate the effect of FRB on muscle atrophy in a streptozotocin (STZ)-induced diabetic rat model. Three groups of Sprague-Dawley rats, namely the control, STZ, and FRB groups, were treated as follows. The diabetic groups (STZ and FRB) were injected intraperitoneally with STZ (40 mg/kg BW), whereas the control group was injected with the vehicle. The STZ and control groups were fed the AIN93M diet, and the FRB group was fed 10% of FRB based on the AIN93M diet. The diabetic groups had reduced muscle size compared to the control group; however, these changes were alleviated in the FRB group. Moreover, the FRB group had a significantly lower expression of FBXO32/Atrogin-1 and TRIM63/MuRF1 (*p* < 0.05) due to blocked NF-κB activation. In conclusion, the anti-inflammatory effect of FRB may be beneficial for ameliorating muscle atrophy in diabetic conditions.

## 1. Introduction

Muscle atrophy occurs when protein degradation rates exceed protein synthesis, which can result from various factors including aging, chronic diseases, nutritional deficiencies, genetics, and physical unloading due to an injury or illness. Atrophied muscles result in an impaired ability to perform daily functions, which increases dependency [1,2] and results in increased health care utilization and costs [3]. Moreover, diabetes leads to a catabolic state with decreased muscle strength, contributing to disease-related morbidity [4], which is an important clinical challenge associated with a decreased quality of life. Therefore, understanding its underlying mechanism(s) is important in preventing these issues.

Chronic inflammation has been implicated in the pathophysiology of diabetes mellitus. Its onset is promoted by hyperglycemia and mediated by oxidative stress [5]. Catabolic conditions leading to muscle atrophy during aging and diabetes are induced in response to oxidative stress and inflammation [6,7]. The streptozotocin (STZ)-induced diabetic rat model has been widely used to screen the potential capabilities of various artificial chemicals, natural products, and pharmacological agents in lowering the chronic complications of diabetes [8]. This animal model is also characterized by muscle atrophy resulting from hyperglycemia and hypoinsulinemia [9,10] and, therefore, serves as an appropriate model to emulate muscle atrophy as a diabetic complication.

Protein degradation, which is one of the important causes of diabetic muscle atrophy, is regulated by two major pathways: the ubiquitin-proteasomal pathway, and the autophagic-lysosomal system [11,12,13]. The ubiquitin-proteasomal pathway is marked by the increased expression of muscle-specific ubiquitin ligases including the F-box only protein 32/atrophy gene 1 (FBXO32/Atrogin-1) and tripartite motif containing 63/muscle-specific RING finger protein 1 (TRIM63/MuRF1), which are regulated by specific transcription factors such as the Forkhead box subfamily O (FOXO) [14,15,16]. Studies have shown that the activation of NF-κB by oxidative stress in muscles, particularly in hyperglycemic conditions, also increases the activity of the ubiquitin-proteasome proteolytic pathway [17,18]. However, this activation is inhibited by several functional compounds such as eicosapentaenoic acid [19] and resveratrol [20], thereby resulting in the stabilization of atrophy. The autophagic-lysosomal pathway serves as a mechanism for degrading and recycling cellular components including bulk cytoplasm, long-lived proteins, and organelles such as mitochondria [11]. Furthermore, this process generates specific markers such as γ-aminobutyric acid type A receptor-associated protein-like 1 (GABARAPL1) and BCL2 interacting protein 3 like (BNIP3L), a marker of mitophagy [4]. The expression profile of these markers has been widely studied to explore the possible mechanisms underlying muscle atrophy.

Nutrition is one of the strategic approaches to maintain muscle health, recover from disease, and prevent and treat muscle weakness in the elderly [21,22]. Rice bran is a byproduct of the rice milling industry that has long been characterized as an excellent source of various nutrients and bioactive compounds. For instance, the active component of rice bran improves the expression of mitochondrial biogenesis and reducing oxidative stress in a mouse model of vascular damage [23]. Meanwhile, phytosterol ferulic acid esters significantly inhibit inducible nitric oxide synthase expression and nitric oxide production, while also significantly inhibiting IκBα degradation, resulting in the inhibition of nuclear factor-kappa B (NF-κB) p65 nuclear translocation [24]. Chronic supplementation of rice bran enzymatic extract also ameliorates atherosclerosis-related oxidative stress and inflammation markers such as interleukine-6 (*Il6*) and tumor necrosis factor α (*Tnfa*) mRNA expressions [25].

The fermented rice bran (FRB) that was used in this study was prepared by the dual fermentation of rice bran using fungi and lactic acid bacteria, which improved the characteristic flavor and total phenolic content of the rice bran itself [26]. This method of preparation of FRB—as a prospective supplement—has been proven to ameliorate certain medical conditions such as hypertension, inflammatory bowel disease, and metabolic syndrome [26,27,28,29,30]. Moreover, the previously defined functions of FRB in reducing oxidative stress, maintaining blood glucose levels, and regulating inflammatory markers were validated in these studies. Since these functions are also important in managing diabetes and preventing muscle atrophy, we hypothesized that the anti-inflammatory and antioxidative effects of FRB play a role in managing muscle atrophy, specifically in the context of diabetic complications.

Hence, we investigated the effect of FRB on muscle atrophy in rats with STZ-induced diabetes. Additionally, we studied the expression of atrogenes including *Fbxo32*/*Atrogin1* and *Trim63*/*Murf1* as well as the associated protein level to validate the ameliorative effects of FRB.

## 2. Materials and Methods

### 2.1. Materials

The components of the AIN-93M standard diet [31] were obtained from Oriental Yeast Co. Ltd. (Tokyo, Japan) and Wako Pure Chemical Industries, Ltd. (Osaka, Japan). FRB, which was prepared by the dual fermentation of rice bran, was kindly supplied by the Sunstar Company (Tokyo, Japan) [26]. STZ was obtained from Wako Pure Chemical Industries. Goat anti-rat IL-1β (R&D Systems, Minneapolis, MN, USA) and rabbit anti-FBXO32/Atrogin-1, TRIM63/MuRF1 (ECM Biosciences, Versailles, KY, USA), ubiquitin, TNF-α, and NF-κB p65 and phosphorylated p65 (Cell Signaling Technology, Danvers, MA, USA) antibodies were used for measuring protein expression.

### 2.2. Animals

Eighteen male Sprague-Dawley rats (eight weeks old) were obtained from Japan SLC (Hamamatsu, Japan), divided into three groups (*n* = 6 per group), and housed in individual cages under controlled conditions (temperature: 23 ± 3 °C; humidity: 50 ± 10%; and a 12/12-h light-dark cycle). After one week of acclimatization, two of the three groups were injected intraperitoneally with STZ (40 mg/kg body weight after being fasted for 6 h prior) as the diabetic groups included the STZ group and the FRB group, and one group—the control group—was injected with the vehicle (0.05 M citrate buffer, pH 4.5, after being fasted for 6 h prior). A dose of 40 mg/kg was deemed suitable as it sufficiently induced diabetic symptoms while maintaining stable conditions in rats. Two days post-treatment, blood was drawn from the tail vein, and the blood glucose level was measured using a glucometer. Rats with fasting blood glucose over 200 mg/dL were considered diabetic and used for subsequent treatments. One rat was found to be unresponsive to the STZ treatment and therefore was excluded from further analysis.

The STZ group and the control group were fed the AIN93M diet (standard diet), and the FRB group was fed 10% of FRB—based on the AIN93M diet—during the entire treatment period (one month). Details of the experimental diets are given in Table 1 [26]. The body weight was measured; additionally, the plasma glucose and insulin levels were analyzed using blood, which was collected from the tail vein. On the thirtieth day, the rats were euthanized after fasting for 6 h. Blood and the gastrocnemius muscles of both hindlimbs were collected. The animal use and care protocols were reviewed and approved by the Animal Research-Animal Care Committee of Tohoku University (Sendai, Japan) according to the Japan governmental legislation (2005). The approved document number of this animal experiment is 2018AgA-014.

### 2.3. Histological Analysis

The gastrocnemius muscle from the left hindlimb was isolated, fixed in 4% formaldehyde, treated with a series concentration of ethanol and toluene, embedded in paraffin, and sectioned into 5-μm slices. The paraffin-embedded sections were then stained with hematoxylin and eosin using standard techniques. Images of the myocyte cross-sectional areas of the muscle were captured using a digital camera at 200x magnification, and 100 fibers per animal (average) were observed. The ImageJ software (https://imagej.nih.gov/ij/) was used to analyze the cross-sectional image of the myocytes.

### 2.4. Blood Glucose Level, Plasma Insulin Level, and Blood Profile

The blood for the estimation of the plasma glucose and insulin levels was drawn from the tail vein. The blood glucose level was measured via a colorimetric method using the glucose C2 test Wako Kit (Wako Pure Chemical Industries, Ltd.), and the insulin level was measured using the rat insulin ELISA Kit (Morinaga, Yokohama, Japan) according to the manufacturers’ instructions. The blood profile was analyzed by Oriental Yeast Co. using an automatic biochemical analyzer (Model 7180 Automatic Analyzer; Hitachi, Ltd., Tokyo, Japan).

### 2.5. Western Blotting

The gastrocnemius muscle was homogenized in phosphate-buffered saline supplemented with a protease inhibitor cocktail (Complete protease inhibitor cocktail; Roche Applied Science, Mannheim, Germany) and a phosphatase inhibitor cocktail (PhosSTOP phosphatase inhibitor cocktail, Roche Applied Science). The concentrations of the protein lysates were measured by spectrophotometry at 565 nm using a protein assay (Bio-Rad, Hercules, CA, USA). The lysate (16 μg) was separated on a 12.5% sodium dodecyl sulfate gel. The separated proteins were then transferred onto an Immobilon-P membrane (Millipore, Billerica, MA, USA). The membrane was incubated in a blocking buffer (10 mM Tris-HCl (pH 7.4), 150 mM NaCl, 0.1% Tween 20, and 5% skim milk or 3% bovine serum albumin) for 1 h. Subsequently, the membrane was incubated overnight with blocking buffer containing primary antibodies. Primary antibodies for FBXO32/Atrogin-1 and TRIM63/MuRF1 were diluted 1:1000 in blocking buffer containing skim milk, whereas antibodies for ubiquitin, IL-1β, TNF-α, and NF-κB p65 and phosphorylated p65 were diluted 1:1000 in blocking buffer containing bovine serum albumin. Immobilon western detection reagent (Millipore) was used with a luminescent image analyzer (LAS-4000 mini; Fujifilm, Tokyo, Japan). Oxidized protein was measured using the Protein Carbonyl Assay Kit (Abcam, Tokyo, Japan) following the manufacturer’s protocol. Fortes et al. [32] reported that Ponceau S staining was a better choice for total protein quantification compared with common housekeeping proteins in STZ-induced diabetes and skeletal muscle hypertrophy experimental models. In this study, we used Coomassie brilliant blue (CBB) staining for total protein quantification as an alternative to the common loading control.

### 2.6. RNA Extraction and Quantitative Reverse Transcriptase (RT)-PCR

The gastrocnemius muscle sample was homogenized using ISOGEN (Nippon Gene Co. Ltd., Tokyo, Japan) to isolate and purify the total RNA, and the quality and quantity of RNA were evaluated by measuring the absorbance at 260 nm and 280 nm on a spectrophotometer. cDNA was then synthesized using the isolated RNA as a template. Briefly, RNA was incubated with 5 μM oligo-dT primer (Hokkaido System Science Co., Sapporo, Japan) and 1 mM dNTP (GE Healthcare, Tokyo, Japan) at 65 °C for 5 min. This denatured RNA mixture was then added to a solution containing RT buffer (50 mM Tris-HCl (pH 8.3), 75 mM KCl, 3 mM MgCl_2_, and 5 mM dithiothreitol), 50 U SuperScriptIII reverse transcriptase (Invitrogen, Carlsbad, CA, USA), and 20 U RNaseOUT RNase inhibitor (Invitrogen). Reverse transcription was carried out at 50 °C for 60 min to produce cDNA. Then, quantitative RT-PCR was performed on CFX Connect Real-Time PCR Detection System (Bio-Rad) using gene-specific primers (Table 2) and the TB Green Premix EX Taq (Takara Bio, Otsu, Japan). Gene expression was normalized to that of eukaryotic elongation factor 1α1 *(Eef1a1)* [30].

### 2.7. Statistical Analysis

Data are presented as the mean + standard error of the means (SEM). Statistical analysis was performed using SigmaPlot version 12.5 (San Jose, CA, USA). Analysis of variance (ANOVA) followed by Tukey’s *post hoc* test was used to analyze the differences between the control, STZ, and FRB groups. The body weights from these three groups were also analyzed using repeated measures ANOVA. All statistical analyses were conducted with a significance level of α = 0.05 (*p* < 0.05).

## 3. Results

### 3.1. Effect of FRB on the Body Weight of Rats with STZ-Induced Diabetes

**Figure 1:**
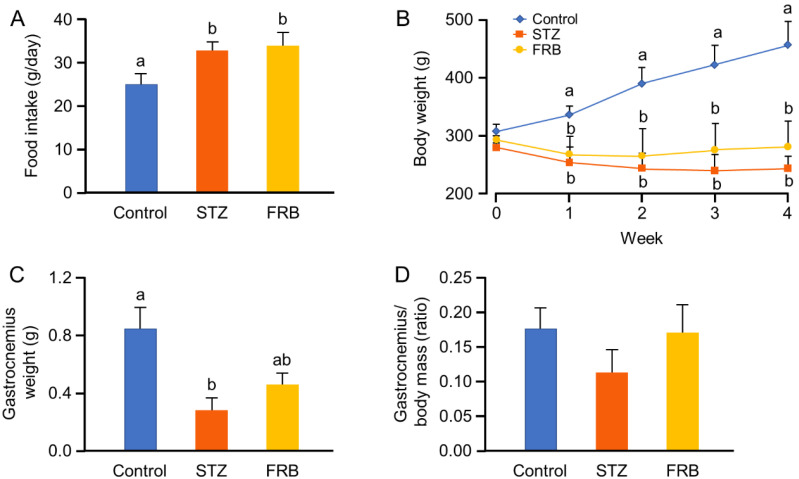
STZ-induced diabetes decreases body weight of rats. (**A**) The average food intake per day; (**B**) body weight changes (analyzed using repeated measures ANOVA); (**C**) gastrocnemius muscle weight; and (**D**) Normalized gastrocnemius weight to body mass. Data are shown as the means + SEM. Significance (*p* < 0.05, *n* = 5–6) among groups is denoted by different letters (a, b).

One week after the STZ injection, the diabetes group showed significantly higher food intake (polyphagia) per day than the control group (Figure 1A). Meanwhile, both diabetic groups showed significantly reduced body weight from the first week onward (Figure 1B). Although a slight inflection of the curve was observed in the FRB group in comparison with the STZ group, there was no significant difference between these two groups. However, the mass of the gastrocnemius muscle in the FRB group was found to be higher than that in the STZ group, although it remained lower than that of the control group (Figure 1C). Interestingly we found no statistical difference between these three groups after the weight of the gastrocnemius muscle was normalized to the rats’ body weight (*p* = 0.51; Figure 1D). Nonetheless, STZ treatment still retained a tendency to decrease the weight of the gastrocnemius muscle, and this tendency was not apparent in the FRB supplemented group. These results indicate that FRB supplementation may directly affect muscle condition in rats, despite the fact that this influence was not apparent in relation to the rats’ overall body weight.

### 3.2. Effect of FRB on the Blood Profile of Rats with STZ-induced Diabetes

**Figure 2:**
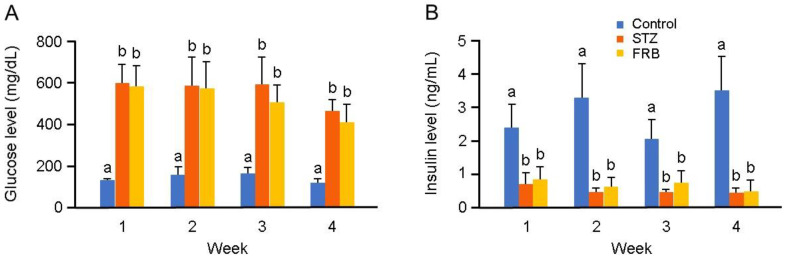
One-month supplementation of FRB could not recover the effect of STZ on hyperglycemia and hypoinsulinemia. (**A**) Blood glucose level; (**B**) plasma insulin level. Data are shown as the means + SEM. Significance (*p* < 0.05, *n* = 5–6) among groups is denoted by different letters (a, b).

The blood glucose and plasma insulin levels in the diabetes groups were significantly different compared to those in the control group (Figure 2A,B). Both the STZ and FRB groups showed a higher level of blood glucose and a lower level of plasma insulin compared to the control group, indicating the efficiency of STZ in inducing diabetes during the one month of treatment.

As shown in Table 3, the profiling of blood samples at the end of the treatment period showed a significant reduction in the levels of total protein, albumin, amylase, triglycerides, and insulin in the STZ and FRB groups compared to those in the control group. In contrast, the levels of high-density lipoprotein (HDL)-cholesterol and glucose were significantly increased in the FRB and STZ groups compared to those in the control group. However, there were no significant differences in these parameters between the STZ and the FRB groups, indicating that FRB supplementation for a period of one month could not recover the devastating effect of STZ on the pancreatic β cells.

### 3.3. Effect of FRB on the Muscle Size of Rats with STZ-induced Diabetes

**Figure 3:**
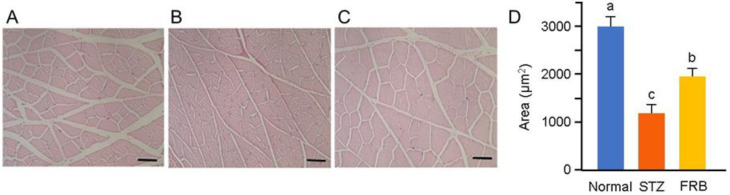
FRB supplementation increased the muscle size in rats with STZ-induced diabetes. Representative myocyte cross-sections of the gastrocnemius muscle of the (**A**) control, (**B**) STZ, and (**C**) FRB groups stained with hematoxylin and eosin. (**D**) The average of the myocyte cross-sectional area. Scale bars represent 50 μm. Significance (*p* < 0.05, *n* = 5–6) among groups is denoted by different letters (a, b, c).

Representative hematoxylin and eosin-stained gastrocnemius muscle cross-sections are shown in Figure 3A–C. The diabetes groups showed a smaller gastrocnemius myocyte cross-sectional area compared to the control group. The FRB group showed an up to 35.26% decrease in the gastrocnemius cross-sectional area, while the STZ group showed an up to 60.52% decrease in the cross-sectional area. The size of the gastrocnemius muscle (Figure 3D) in the FRB group was significantly larger than that in the STZ group, but was still lower than the control group. This suggests that FRB was able to partially attenuate the devastating effect of STZ-induced diabetes in rat muscles.

### 3.4. Effect of FRB on Diabetic Muscle Regulatory Genes in Rats with STZ-induced Diabetes

**Figure 4:**
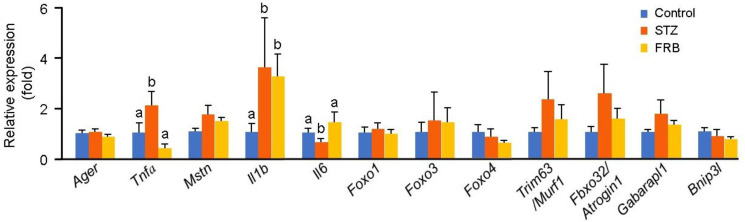
FRB supplementation influenced the expression of muscle regulatory genes. Data are shown as the means + SEM. Significance (*p* < 0.05, *n* = 5) among groups is denoted by different letters (a, b).

To evaluate the effect of FRB on diabetic muscles, the mRNA expression of diabetic muscle regulatory genes was analyzed (Figure 4). There were no significant differences in the mRNA expression levels of the stimulators of protein degradation in hyperglycemic conditions) receptor of the advanced glycation end product (*Ager*) and myostatin (*Mstn*)), although a slight increase was seen in the level of *Mstn* in the diabetic groups (*p* = 0.11). Furthermore, the expression of *Tnfa* was significantly decreased in the FRB group compared to that in the STZ group, and its level was comparable with that in the control group. The expression of interleukin 1 *beta* (*Il1b*) in the diabetic groups was higher than that in the control group. Interestingly, the expression of *Il6* in the FRB group was elevated significantly compared to that in the STZ group. The expressions of *Foxo1* (*p* = 0.79), *Foxo3* (*p* = 0.89), *Foxo4* (*p* = 0.38), *Trim63*/*Murf1* (*p* = 0.48), *Fbxo32*/*Atrogin1* (*p* = 0.30), *Bnip3l* (*p* = 0.36), and *Gabarapl1* (*p* = 0.37) did not change following FRB supplementation.

### 3.5. Effect of FRB on the Level of Oxidized and Ubiquitinated Proteins in the Muscles of Rats with STZ-Induced Diabetes

**Figure 5:**
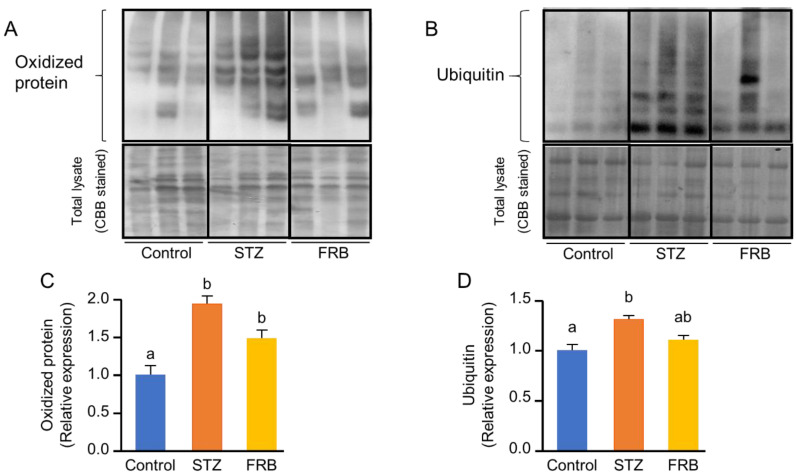
The effect of FRB supplementation on the level of oxidized proteins and ubiquitinated proteins. Representative blots of the (**A**) oxidized proteins and (**B**) ubiquitinated proteins, and the quantification of the (**C**) oxidized proteins and (**D**) ubiquitinated proteins. Data are shown as the means + SEM. Significance (*p* < 0.05, *n* = 5) among groups is denoted by different letters (a, b, ab).

Hyperglycemia-induced oxidative stress and chronic inflammation in diabetes lead to the activation of the ubiquitin-proteasomal pathway, which facilitates protein degradation in the muscle. Although there was no significant difference between the STZ and FRB groups, FRB supplementation caused a slight decrease in the level of oxidized proteins (Figure 5A,B). Meanwhile, FRB supplementation had a moderate effect on the lowering of the level of ubiquitinated proteins after STZ treatment (Figure 5C,D).

### 3.6. Effect of FRB on the Protein Degradation Markers in the Muscles of Rats with STZ-induced Diabetes

**Figure 6:**
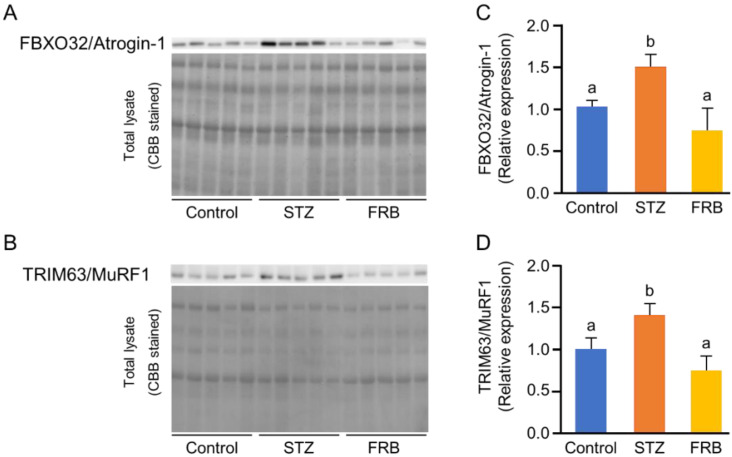
FRB decreased the expression of muscle-specific ubiquitin ligases. Representative blots of (**A**) FBXO32/Atrogin-1 and (**B**) TRIM63/MuRF1. The quantification of (**C**) FBXO32/Atrogin-1 and (**D**) TRIM63/MuRF1. Data are shown as the means + SEM. Significance (*p* < 0.05, *n* = 5) among groups is denoted by different letters (a, b).

Next, we analyzed the protein levels of FBXO32/Atrogin-1 and TRIM63/MuRF1, the markers of muscle-specific ubiquitin ligases, to confirm the effect of FRB on atrophy in diabetic muscles. The results showed that FRB significantly reduced the relative expression of both FBXO32/Atrogin-1 and TRIM63/MuRF1 compared to that in the STZ group (Figure 6A–D).

### 3.7. Effect of FRB on the Phosphorylation of NF-κB p65 in the Muscles of Rats with STZ-induced Diabetes

**Figure 7:**
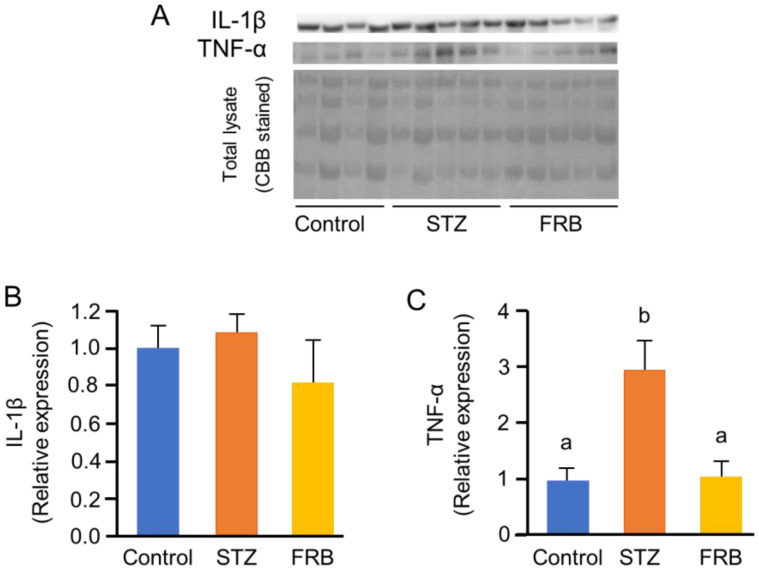
FRB decreased the expression of inflammatory cytokines. Representative blots of (**A**) IL-1β and TNF-α, and quantification of (**B**) IL-1β and (**C**) TNF-α. Data are shown as the means + SEM. Significance (*p* < 0.05, *n* = 4–5) among groups is denoted by different letters (a, b).

**Figure 8:**
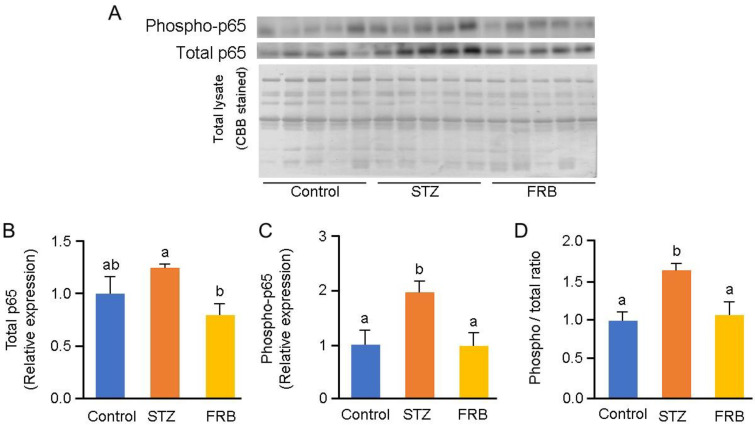
FRB inhibits NF-κB activation. Representative blots of (**A**) phosphorylated p65 (phospho-p65) and total p65, and the quantification of (**B**) total p65, (**C**) phospho-p65, and (**D**) phospho-p65/p65 ratio. Data are shown as the means + SEM. Significance (*p* < 0.05, *n* = 5) among groups is denoted by different letters (a, b, ab).

Next, we estimated the effect of FRB on the relative expression of IL-1β, TNF-α (Figure 7A), NF-κB p65, and its phosphorylated form, phospho-p65 (Figure 8A). While the results showed no significant differences in the protein level of IL-1β (*p* = 0.51; Figure 7B), the relative expression of TNF-α in the FRB group was lower than that in the STZ group (*p* < 0.05; Figure 7C).

Additionally, FRB reduced the relative expression of NF-κB p65 and phospho-p65 (*p* < 0.05; Figure 8B,C) as well as the phospho-p65/p65 ratio (*p* < 0.05; Figure 8D). These results indicate that the primary loss of the insulin response caused by STZ treatment can promote proinflammatory signaling in the muscle, particularly via TNF-α, which further activates the transcription factor NF-κB. It also suggests that FRB supplementation prevents the activation of this pathway.

## 4. Discussion

The STZ-induced diabetic rat model has been shown to represent conditions associated with type 1 diabetes, which is characterized by muscle atrophy—due to diabetic glucotoxicity—and symptoms such as hyperglycemia, hypoinsulinemia, hyperurination, polyphagia, polydipsia, glucosuria, and weight loss [33]. In the present study, we showed that diabetic complications—induced upon an injection of 40 mg of STZ per kg body weight—resulted in muscle atrophy in our model, which was characterized by polyphagia and resulted in decreased plasma insulin levels and increased blood glucose levels, followed by a reduction in the body weight. Moreover, the blood profile of the diabetic groups in this study showed that the destruction of the pancreatic β cells by STZ resulted in the generation of a hyperglycemic condition, which could lead to the development of oxidative stress and altered conditions such as hepato-renal inflections and lipid mobilization; this was similar to the studies of Rai et al. [34].

Diabetes mellitus directly leads to muscle atrophy via the activation of enzymes involved in the ubiquitin-proteasome system, which could be induced by another pathway [18,35], and may contribute to muscle protein degradation by removing contractile proteins and organelles, resulting in the shrinkage of muscle mass and myofiber size [12,36]. This study showed that the diabetic complications caused by STZ-induced glucotoxicity decreased body weight, which was followed by shrinkage of muscle fibers in the diabetic groups. Although FRB supplementation did not prevent body weight loss, one month of FRB supplementation affected other symptoms commonly associated with STZ treatment. FRB supplementation partially reversed the loss of gastrocnemius muscle mass as well as significantly ameliorated rats’ muscle shrinkage.

Hyperglycemic conditions induce the diabetes-related genes coding for cell surface stimulators. It has been shown that *Ager,* which regulates glucose homeostasis [37], and *Mstn*, which regulates myogenesis, are highly expressed in diabetic conditions [38], whereas *Tnfa* is highly expressed in hypoinsulinemia [39]. These factors contribute to the development of oxidative stress in diabetic organs. In this study, we showed that FRB supplementation significantly reduced *Tnfa* mRNA expression.

FOXO family proteins play a vital role in the development of muscle atrophy by activating the ubiquitin-mediated proteolysis pathways including the regulation of FBXO32/Atrogin-1 and TRIM63/MuRF1 [40], and dependently regulate BNIP3L and GABARAPL1 in a STZ-diabetes model [4]. The present study exhibited that FRB supplementation reduced the protein levels of both FBXO32/Atrogin-1 and TRIM63/MuRF1, two muscle-specific E3 ubiquitin ligases, in the diabetic muscle. Since the level of ubiquitinated proteins was also found to decrease in the FRB group compared to that in the STZ group, the FRB-supplemented diet was suggested to be able to inhibit the ubiquitin-mediated proteolysis pathway, thereby limiting the expression of FBXO32/Atrogin-1 and TRIM63/MuRF1. Thus, the reduced expression of these specific protein levels in the FRB group compared to that in the STZ group confirmed the beneficial effects of FRB supplementation in regulating the ubiquitin-mediated proteolysis pathways.

Both type 1 and type 2 diabetic conditions are associated with significantly elevated levels of serum TNF-α and exhibit a positive correlation with insulin resistance [41]. Additionally, the critical role of reactive oxygen species induced TNF-α in the activation of NF-κB—a key step in the overall processes that mediate muscle atrophy—has been reported [42]. TNF-α and IL-1β, as products of the NF-κB pathway, can also serve as activators of NF-κB, thereby initiating a positive feedback loop that enhances the expression of NF-κB and exacerbates muscle degradation [43]. FBXO32/Atrogin-1 was proven to be overexpressed in response to TNF-α treatment in C2C12 cells [44]. NF-κB activated by TNF-α was also found to upregulate TRIM63/MuRF1 [45]. In addition to having the capacity to decrease TNF-α protein levels and the tendency to reduce the oxidized proteins, FRB supplementation was also shown to reduce the NF-κB phospho-p65/p65 ratio in comparison with STZ treatment alone, further confirming that FRB supplementation reduced the activation of the NF-κB pathway that occurred due to STZ-induced diabetes. This process will consequently inhibit the production of FBXO32/Atrogin-1 and TRIM63/MuRF1 in diabetic rats.

Here, we have shown the effect of FRB by estimating the expression of the different markers involved in the proteolysis pathways. Although the protein synthesis pathway is also an important factor in muscle atrophy, this pathway was not included in this study. Hence, further studies are needed to elucidate the detailed mechanism by which the FRB components prevent muscle atrophy during aging, diabetes, or other chronic diseases.

## 5. Conclusions

The results of this study indicate that FRB supplementation reduced muscle atrophy in a diabetic muscle atrophy model as FRB supplementation partially prevented muscle size reduction in diabetic conditions. Moreover, FRB supplementation reduced the expression of FBXO32/Atrogin-1 and TRIM63/MuRF1 by inhibiting the activation of NF-κB and interfered with the TNF-α feedback loop that may exacerbate its activation. The findings of this study suggest that the functional components in FRB act as anti-inflammatory agents, which may ultimately result in a protective effect with regard to muscle atrophy in diabetic complications, thereby indicating the potential therapeutic applications of FRB in aging and diabetes.

## Figures and Tables

**Table 1:** Details of the diet formula used in the present study.

Components (g/kg)	Control Group	Diabetes Groups	
		STZ	FRB
	Standard Diet		10% FRB
*Tert*-Butylhydroquinone	0.008		0.0072
l-Cystine	1.8		1.62
Choline bitartrate	2.5		2.25
Vitamin mixture (AIN-93G)	10		9
Mineral mixture (AIN-93M)	35		31.5
Soybean oil	40		36
Cellulose	50		45
Sucrose	100		90
Casein	140		126
Cornstarch	620.7		558.623
Fermented rice bran	0		100

STZ: streptozotocin, FRB: fermented rice bran.

**Table 2:** List of the nucleotide sequence of gene-specific primers.

Gene Name	Forward Primer (5′-3′)	Reverse Primer (5′- 3′)
*Ager*	GAAACCGGTGATGAAGGACAAG	TCGAGTCTGGGTTGTCGTTTT
*Tnfa*	GACGTGGAACTGGCAGAAGAG	TCTGGAAGCCCCCCATCT
*Mstn*	TACCATGCCTACCGAGTCTGA	CAGCTGGGCCTTTACCACTT
*Il1b*	CTGTGTCTTTCCCGTGGACC	CAGCTCATATGGGTCCGACA
*Il6*	AGAGGAGACTTCACAGAGGATACCA	AATCAGAATTGCCATTGCACAAC
*Foxo1*	CTAGGAGTTAGTGAGCAGGCAA	CTGCCAAGTCTGACGAAAGG
*Foxo3*	GGGGAACTTCACTGGTGCTAA	AGTTTGAGGGTCTGCTTTGC
*Foxo4*	AGGCTCCTACACTTCTGTTACTGG	CTTCAGTAGGAGATGCAAGCACAG
*Trim63/Murf1*	CCTGTGAAGTTGCCCCCTTA	GCTGTTTTCCTTGGTCACTCG
*Fbxo32/Atrogin1*	ACTAAGGAGCGCCATGGAT	CAGGGATGTGAGCTGTGACTT
*Gabarapl1*	ACAACACGATCCACAGCCTT	TCTGCCCAAAGTGACGAGAG
*Bnip3l*	GCTGGCCTCAACAGTTCCTG	TGGATGGAAGACGAGGAAGGA
*Eef1a1*	GATGGCCCCAAATTCTTGAAG	GGACCATGTCAACAATGGCAG

**Table 3:** Blood profile of STZ-induced diabetes after one-month treatment.

Parameter	Unit	Group		
		Control	STZ	FRB
Total protein	g/dL	6.97 ± 0.51 ^a^	5.62 ± 0.38 ^b^	5.98 ± 0.72 ^b^
Albumin	g/dL	4.58 ± 0.35 ^a^	3.62 ± 0.22 ^b^	3.68 ± 0.45 ^b^
Amylase	IU/L	2308.33 ± 204 ^a^	1045.50 ± 326 ^b^	1329.00 ± 399 ^b^
Triglyceride	mg/dL	359.50 ± 139 ^a^	69.17 ± 30.2 ^b^	130.00 ± 101 ^b^
HDL-cholesterol	mg/dL	33.17 ± 3.25 ^a^	43.67 ± 8.66 ^b^	44.40 ± 10.30 ^b^
Glucose	mg/dL	153.83 ± 11.36 ^a^	680.00 ± 125.65 ^b^	614.00 ± 68.7 ^b^
Insulin	ng/mL	4.42 ± 0.99 ^a^	0.44 ± 0.13 ^b^	0.51 ± 0.29 ^b^

STZ: streptozotocin, FRB: fermented rice bran, HDL: high-density lipoprotein. Data are shown as the means ± SEM and were analyzed using ANOVA followed by Tukey’s post hoc test. Significance (*p* < 0.05, *n* = 5–6) among groups is denoted by different letters (a, b).

## References

[1] Faulkner J.A., Larkin L.M., Claflin D.R., Brooks S.V. (2007). Age-related changes in the structure and function of skeletal muscles. Clin. Exp. Pharm. Physiol..

[2] Matsumoto H., Hagino H., Wada T., Kobayashi E. (2016). Locomotive syndrome presents a risk for falls and fractures in the elderly Japanese population. Osteoporos. Sarcopenia.

[3] Beaudart C., Rizzoli R., Bruyère O., Reginster J.-Y., Biver E. (2014). Sarcopenia: Burden and challenges for public health. Arch. Public Health.

[4] O’Neill B.T., Bhardwaj G., Penniman C.M., Krumpoch M.T., Beltran P.A.S., Klaus K., Poro K., Li M., Pan H., Dreyfuss J.M. (2019). FoxO transcription factors are critical regulators of diabetes-related muscle atrophy. Diabetes.

[5] Oguntibeju O.O. (2019). Type 2 diabetes mellitus, oxidative stress and inflammation: Examining the links. Int. J. Physiol. Pathophysiol. Pharm..

[6] Moylan J.S., Reid M.B. (2007). Oxidative stress, chronic disease, and muscle wasting. Muscle Nerve.

[7] Meng S.-J., Yu L.-J. (2010). Oxidative stress, molecular inflammation and sarcopenia. Int. J. Mol. Sci..

[8] Wu J., Yan L.-J. (2015). Streptozotocin-induced type 1 diabetes in rodents as a model for studying mitochondrial mechanisms of diabetic β cell glucotoxicity. Diabetes Metab. Syndr. Obes. Targets.

[9] Ono T., Takada S., Kinugawa S., Tsutsui H. (2015). Curcumin ameliorates skeletal muscle atrophy in type 1 diabetic mice by inhibiting protein ubiquitination. Exp. Physiol..

[10] Hirata Y., Nomura K., Senga Y., Okada Y., Kobayashi K., Okamoto S., Minokoshi Y., Imamura M., Takeda S., Hosooka T. (2019). Hyperglycemia induces skeletal muscle atrophy via a WWP1/KLF15 axis. JCI Insight.

[11] Sandri M. (2013). Protein breakdown in muscle wasting: Role of autophagy-lysosome and ubiquitin-proteasome. Int. J. Biochem. Cell Biol..

[12] Bonaldo P., Sandri M. (2013). Cellular and molecular mechanisms of muscle atrophy. Dis. Model. Mech..

[13] Schreiber A., Peter M. (2014). Substrate recognition in selective autophagy and the ubiquitin–proteasome system. Biochim. Biophys. Acta BBA Mol. Cell Res..

[14] Bodine S.C., Baehr L.M. (2014). Skeletal muscle atrophy and the E3 ubiquitin ligases MuRF1 and MAFbx/atrogin-1. Am. J. Physiol. Endocrinol. Metab..

[15] Sandri M. (2008). Signaling in muscle atrophy and hypertrophy. Physiology.

[16] Gomes M.D., Lecker S.H., Jagoe R.T., Navon A., Goldberg A.L. (2001). Atrogin-1, a muscle-specific F-box protein highly expressed during muscle atrophy. Proc. Natl. Acad. Sci. USA.

[17] Eley H.L., Tisdale M.J. (2007). Skeletal muscle atrophy, a link between depression of protein synthesis and increase in degradation. J. Biol. Chem..

[18] Roy B. (2013). Biomolecular basis of the role of diabetes mellitus in osteoporosis and bone fractures. World J. Diabetes.

[19] Whitehouse A.S., Tisdale M.J. (2003). Increased expression of the ubiquitin–proteasome pathway in murine myotubes by proteolysis-inducing factor (PIF) is associated with activation of the transcription factor NF-κB. Br. J. Cancer.

[20] Wyke S.M., Russell S.T., Tisdale M.J. (2004). Induction of proteasome expression in skeletal muscle is attenuated by inhibitors of NF-κB activation. Br. J. Cancer.

[21] Magne H., Auzeloux I.S., Rémond D., Dardevet D. (2013). Nutritional strategies to counteract muscle atrophy caused by disuse and to improve recovery. Nutr. Res. Rev..

[22] Jiménez J.D.B., Lluch G.L., Martínez I.S., Jiménez A.M., Bies E.R., Navas P. (2011). Sarcopenia, implications of physical exercise in its pathophysiology. prevention and treatment. Rev. Med. Deporte.

[23] Ternero C.P., Werner C.M., Nickel A.G., Herrera M.D., Motilva M.-J., Böhm M., Sotomayor M.A.d., Laufs U. (2017). Ferulic acid, a bioactive component of rice bran, improves oxidative stress and mitochondrial biogenesis and dynamics in mice and in human mononuclear cells. J. Nutr. Biochem..

[24] Islam M.S., Nagasaka R., Ohara K., Hosoya T., Ozaki H., Ushio H., Hori M. (2011). Biological abilities of rice bran-derived antioxidant phytochemicals for medical therapy. Curr. Top. Med. Chem..

[25] Ternero C.P., Pulgarin B.B., Sotomayor M.A.d., Herrera M.D. (2016). Atherosclerosis-related inflammation and oxidative stress are improved by rice bran enzymatic extract. J. Funct. Foods.

[26] Alauddin M., Shirakawa H., Koseki T., Kijima N., Budijanto S., Islam J., Goto T., Komai M. (2016). Fermented rice bran supplementation mitigates metabolic syndrome in stroke-prone spontaneously hypertensive rats. BMC Complement. Altern. Med..

[27] Yu Y., Zhang J., Wang J., Sun B. (2019). The anti-cancer activity and potential clinical application of rice bran extracts and fermentation products. RSC Adv..

[28] Ardiansyah N., Shirakawa H., Sugita Y., Koseki T., Komai M. (2010). Anti-metabolic syndrome effects of adenosine ingestion in stroke-prone spontaneously hypertensive rats fed a high-fat diet. Br. J. Nutr..

[29] Islam J., Koseki T., Watanabe K., Ardiansyah A., Budijanto S., Oikawa A., Alauddin M., Goto T., Aso H., Komai M. (2017). Dietary supplementation of fermented rice bran effectively alleviates dextran sodium sulfate-induced colitis in mice. Nutrients.

[30] Shibayama J., Kuda T., Shikano A., Fukunaga M., Takahashi H., Kimura B., Ishizaki S. (2018). Effects of rice bran and fermented rice bran suspensions on caecal microbiota in dextran sodium sulphate-induced inflammatory bowel disease model mice. Food Biosci..

[31] Reeves P.G., Nielsen F.H., Fahey G.C. (1993). AIN-93 purified diets for laboratory rodents: Final report of the american institute of nutrition Ad Hoc writing committee on the reformulation of the AIN-76A rodent diet. J. Nutr..

[32] Fortes M.A.S., Nassr G.N.M., Vitzel K.F., Pinheiro C.H.d.J., Newsholme P., Curi R. (2016). Housekeeping proteins: How useful are they in skeletal muscle diabetes studies and muscle hypertrophy models?. Anal. Biochem..

[33] Fischer Y.W., Garyantes T. Improving the Reliability and Utility of Streptozotocin-Induced Rat Diabetic Model. https://www.hindawi.com/journals/jdr/2018/8054073/.

[34] Rai S., Hajam Y.A., Basheer M. (2016). Biochemical and histopathological inflections in hepato-renal tissues of streptozotocin (STZ) induced diabetic male rats: Impact of exogenous melatonin administration. J. Clin. Res. Bioeth..

[35] Wang X., Hu Z., Hu J., Du J., Mitch W.E. (2006). Insulin resistance accelerates muscle protein degradation: Activation of the ubiquitin-proteasome pathway by defects in muscle cell signaling. Endocrinology.

[36] Kalyani R.R., Corriere M., Ferrucci L. (2014). Age-related and disease-related muscle loss: The effect of diabetes, obesity, and other diseases. Lancet Diabetes Endocrinol..

[37] Junior D.C.P., Silva K.S., Michalani M.L., Yonamine C.Y., Esteves J.V., Fabre N.T., Thieme K., Catanozi S., Okamoto M.M., Seraphim P.M. (2018). Advanced glycation end products-induced insulin resistance involves repression of skeletal muscle GLUT4 expression. Sci. Rep..

[38] Coleman S.K., Rebalka I.A., D’Souza D.M., Deodhare N., Desjardins E.M., Hawke T.J. (2016). Myostatin inhibition therapy for insulin-deficient type 1 diabetes. Sci. Rep..

[39] Federici M., Hribal M.L., Menghini R., Kanno H., Marchetti V., Porzio O., Sunnarborg S.W., Rizza S., Serino M., Cunsolo V. (2005). Timp3 deficiency in insulin receptor–haploinsufficient mice promotes diabetes and vascular inflammation via increased TNF-α. J. Clin. Investig..

[40] Sandri M., Sandri C., Gilbert A., Skurk C., Calabria E., Picard A., Walsh K., Schiaffino S., Lecker S.H., Goldberg A.L. (2004). Foxo transcription factors induce the atrophy-related ubiquitin ligase atrogin-1 and cause skeletal muscle atrophy. Cell.

[41] Alzamil H. (2020). Elevated serum TNF- *α* is related to obesity in type 2 diabetes mellitus and is associated with glycemic control and insulin resistance. J. Obes..

[42] Thoma A., Lightfoot A.P., Xiao J. (2018). NF-kB and inflammatory cytokine signalling: Role in skeletal muscle atrophy. Muscle Atrophy.

[43] Li H., Malhotra S., Kumar A. (2008). Nuclear factor-kappa B signaling in skeletal muscle atrophy. J. Mol. Med..

[44] Yuan L., Han J., Meng Q., Xi Q., Zhuang Q., Jiang Y., Han Y., Zhang B., Fang J., Wu G. (2015). Muscle-specific E3 ubiquitin ligases are involved in muscle atrophy of cancer cachexia: An in vitro and in vivo study. Oncol. Rep..

[45] Cai D., Frantz J.D., Tawa N.E., Melendez P.A., Oh B.-C., Lidov H.G.W., Hasselgren P.-O., Frontera W.R., Lee J., Glass D.J. (2004). IKKβ/NF-κB activation causes severe muscle wasting in mice. Cell.

